# Supplementary material for: Screening by changes in stereotypical behavior during cell motility
Source: Sci Rep. 2019 Jun 19;9:8784. doi: 10.1038/s41598-019-45305-w (PMC6584642; doi:10.1038/s41598-019-45305-w)
Supplement: Supplementary file 1 — Supplementary Information [file 41598_2019_45305_MOESM1_ESM.pdf]

Supplementary Information for
Screening by changes in stereotypical behavior during cell motility

Luke Tweedy, Patrick Witzel, Doris Heinrich, Robert H. Insall and Robert G. Endres

1 Supplementary figures

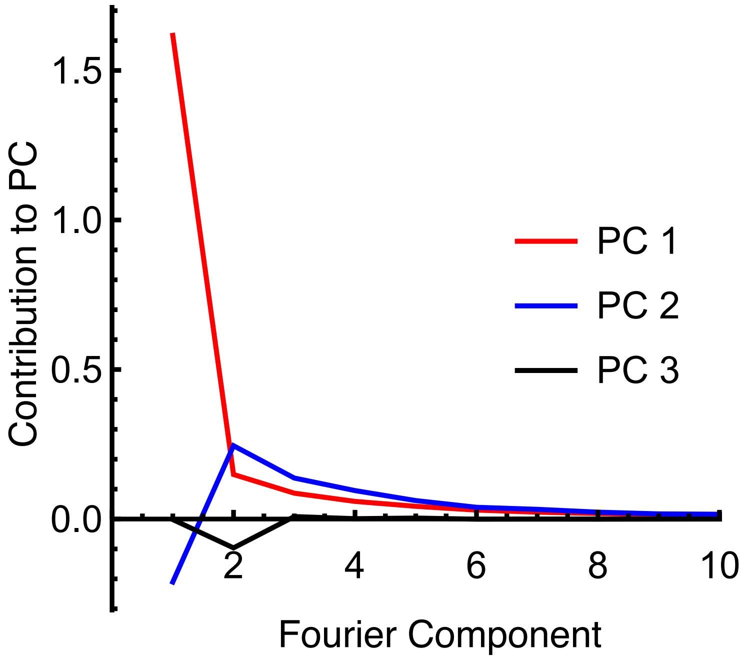

Figure S1: **Power spectrum of the first ten Fourier components for the first 3 principal components.** The largest contributions to each principal component (PC) come from the earliest few Fourier components (FC), though magnitudes do not strictly diminish, for example PC 3 largely aligns with FC 2 and has a minimal contribution from FC 1.

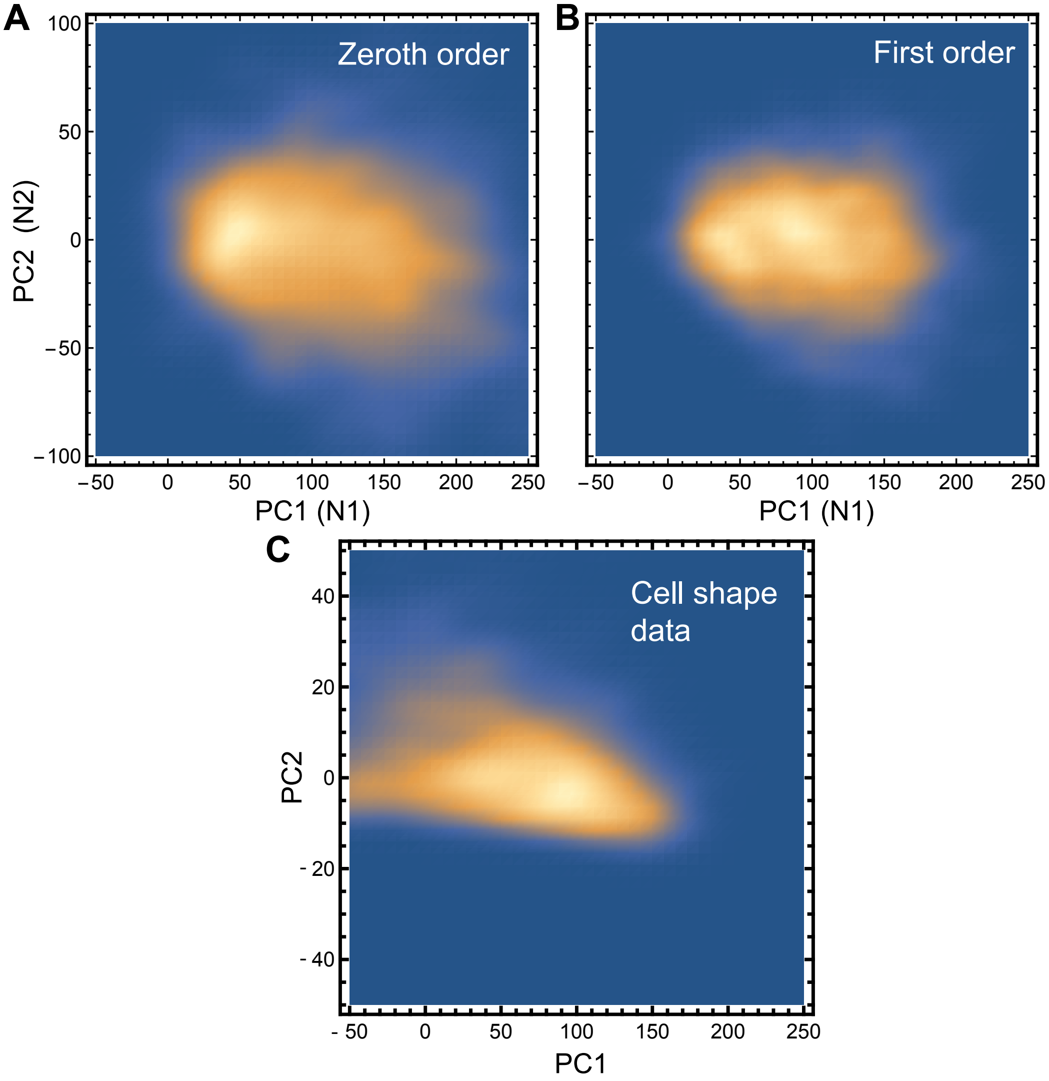

Figure S2: **First order formulation of MaxCal tightens distribution of observed values.** Distribution of observed points in zeroth-order (A) and first-order (B) Maximum Caliber simulations, and in experimentally observed cell shape data (C). In both simulated cases, simulations are run for 5000 timesteps before sampling the distribution. The zeroth-order transition likelihoods do not depend on the current values of N1 and N2. The first order transition likelihoods do depend on N1 and N2 as described in the main text, tightening the overall distribution.

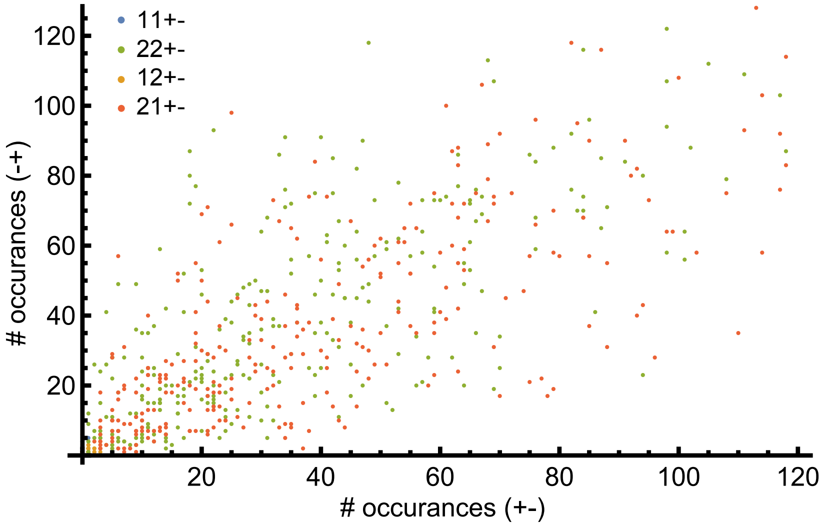

Figure S3: **Similar commonality of trajectory observations under time reversal.** The number of observations within each cell trajectory of each correlated movement that was simplified by time reversal. This figure uses the untreated AX3 data. The four observations shown are (11+-, 11-+), (22+-, 22-+), (12+-, 12-+) and (21+-, 21-+). 11+- and 22+- are both exceptionally rare, and are clustered at the bottom left. Note that none of the four sets drift far from x=y, showing that their likelihoods remain similar under time reversal.

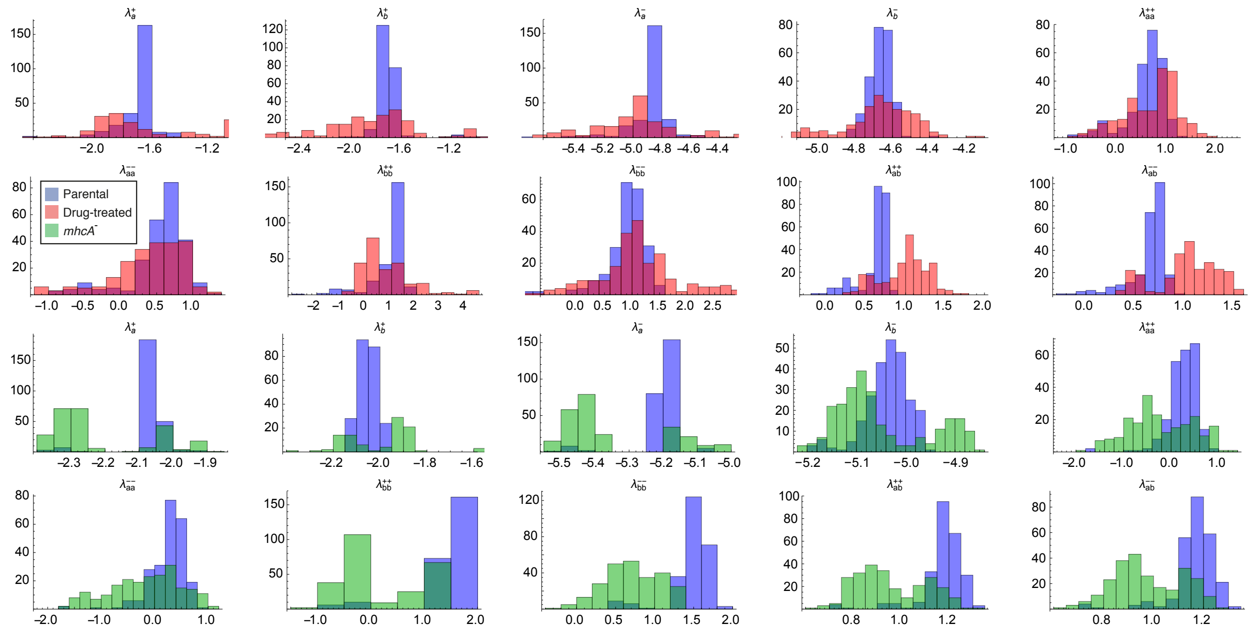

Figure S4: **Distributions of Lagrange multipliers not shown in the main text.** Distribution of values of the Lagrange multipliers in the full partition function of the MaxCal model, accompanying main text Fig. 4. The values are estimated on samples of 5% of the full data set, sampling with replacement. Parent distributions are shown in blue alongside their experimental pairing, with the drug-treated condition in red and the myosin heavy chain knockout in green.

2 Supplementary equations and discussion

2.1 Maximum caliber model

Maximum caliber (MaxCal) is a method for inferring a minimal model and its parameters from a set of data. It is similar to maximum entropy (MaxEnt) methods in equilibrium physics, but instead applies to dynamical systems. As such, it can be used to interrogate systems that are far from equilibrium.

In the main text, we provided the partition function for a single species model only, as a guide to the method. Here, we detail the full partition function $Q(N_1, N_2)$ of the MaxCal model we use to reach our conclusions. The partition function takes the form:

$$
\begin{aligned}
Q(N_1,N_2) = \gamma^{+}_{1}\gamma^{+}_{2}\Big[& \left(\gamma^{+}_{1}\gamma^{++}_{1\,1}\gamma^{++}_{1\,2}+1+(N_1+1)\gamma^{-}_{1}\gamma^{+-}_{1\,1}\gamma^{-+}_{1\,2}\right) \\
& \left(\gamma^{+}_{2}\gamma^{++}_{1\,2}\gamma^{++}_{2\,2}+1+(N_2+1)\gamma^{-}_{2}\gamma^{+-}_{1\,2}\gamma^{+-}_{2\,2}\right)\Big] + \\
\gamma^{+}_{1}\Big[& \left(\gamma^{+}_{1}\gamma^{++}_{1\,1}+1+(N_1+1)\gamma^{-}_{1}\gamma^{+-}_{1\,1}\right)\left(\gamma^{+}_{2}\gamma^{++}_{1\,2}+1+N_2\gamma^{-}_{2}\gamma^{+-}_{1\,2}\right)\Big] + \\
N_2\gamma^{+}_{1}\gamma^{-}_{2}\Big[& \left(\gamma^{+}_{1}\gamma^{++}_{1\,1}\gamma^{+-}_{1\,2}+1+(N_1+1)\gamma^{-}_{1}\gamma^{+-}_{1\,1}\gamma^{--}_{1\,2}\right) \\
& \left(\gamma^{+}_{2}\gamma^{++}_{1\,2}\gamma^{+-}_{2\,2}+1+(N_2-1)\gamma^{-}_{2}\gamma^{+-}_{1\,2}\gamma^{--}_{2\,2}\right)\Big] + \\
\gamma^{+}_{2}\Big[& \left(\gamma^{+}_{1}\gamma^{++}_{1\,2}+1+N_1\gamma^{-}_{1}\gamma^{-+}_{1\,2}\right)\left(\gamma^{+}_{2}\gamma^{++}_{2\,2}+1+(N_2+1)\gamma^{-}_{2}\gamma^{+-}_{2\,2}\right)\Big] + \\
\Big[& \left(\gamma^{+}_{1}+1+(N_1)\gamma^{-}_{1}\right)\left(\gamma^{+}_{2}+1+N_2\gamma^{-}_{2}\right)\Big] + \\
N_2\gamma^{-}_{2}\Big[& \left(\gamma^{+}_{1}\gamma^{+-}_{1\,2}+1+N_1\gamma^{-}_{1}\gamma^{--}_{1\,2}\right)\left(\gamma^{+}_{2}\gamma^{+-}_{2\,2}+1+(N_2-1)\gamma^{-}_{2}\gamma^{--}_{2\,2}\right)\Big] \\
N_1\gamma^{-}_{1}\gamma^{+}_{2}\Big[& \left(\gamma^{+}_{1}\gamma^{+-}_{1\,1}\gamma^{-+}_{1\,2}+1+(N_1-1)\gamma^{-}_{1}\gamma^{--}_{1\,1}\gamma^{-+}_{1\,2}\right) \\
& \left(\gamma^{+}_{2}\gamma^{-+}_{1\,2}\gamma^{++}_{2\,2}+1+(N_2+1)\gamma^{-}_{2}\gamma^{--}_{1\,2}\gamma^{+-}_{2\,2}\right)\Big] + \\
N_1\gamma^{-}_{1}\Big[& \left(\gamma^{+}_{1}\gamma^{+-}_{1\,1}+1+(N_1-1)\gamma^{-}_{1}\gamma^{--}_{1\,1}\right)\left(\gamma^{+}_{2}\gamma^{-+}_{1\,2}+1+N_2\gamma^{-}_{2}\gamma^{--}_{1\,2}\right)\Big] + \\
N_1N_2\gamma^{-}_{1}\gamma^{-}_{2}\Big[& \left(\gamma^{+}_{1}\gamma^{+-}_{1\,1}\gamma^{--}_{1\,2}+1+(N_1-1)\gamma^{-}_{1}\gamma^{--}_{1\,1}\gamma^{--}_{1\,2}\right) \\
& \left(\gamma^{+}_{2}\gamma^{-+}_{1\,2}\gamma^{+-}_{2\,2}+1+(N_2-1)\gamma^{-}_{2}\gamma^{--}_{1\,2}\gamma^{--}_{2\,2}\right)\Big],
\end{aligned}
\tag{1}
$$

where N_1 and N_2 are the rounded values of our first two principal components of shape, and for any x, y we use the notation $\gamma^{y}_{x} = \exp(\lambda^{\,c\,d}_{\,1\,2})$. The values of the Lagrange multiplers λ_n relate to the average rates with which specific patterns of shape change are observed in the data. These shape changes are combinations of increases and decreases in a shape component, both within and across PCs, for example an increase in PC 1 followed by a decrease in PC 2. The expression that relates λ_n to these observed data is:

$$
\begin{aligned}
\langle S_n \rangle &= \frac{\partial \ln Q}{\partial \lambda_n} \\
&= \frac{1}{Q}\frac{\partial Q}{\partial \lambda_n}.
\end{aligned}
\tag{2}
$$

where $\langle S_n \rangle$ is the observed rate of the pattern n. Eq. 2 gives us a method for finding a set of simultaneous equations for the values of our Lagrange multipliers λ_n. Using our partition function, these equations take the following forms:

$$
\begin{aligned}
\langle S^+_1 \rangle = \frac{\gamma^+_1}{Q} \Big[1 + N_2\gamma^-_2 + \gamma^+_2 + N_1\gamma^{+-}_{11}\gamma^-_1\big(1 + (N_2-1)\gamma^{--}_{12}\gamma_2 + \gamma^{-+}_{12}\gamma^+_2\big) + \\
\gamma^{++}_{11}\gamma^+_1\big(1 + N_2\gamma^{+-}_{12}\gamma^-_2 + \gamma^{++}_{12}\gamma^+_2\big) + \\
N_2\gamma^{+-}_{12}\gamma^-_2\big(1 + (N_2-1)\gamma^{--}_{22}\gamma^-_2 + \gamma^{+-}_{22}\gamma^+_2\big) + \\
\big(1 + (N_1+1)\gamma^{+-}_{11}\gamma^-_1 + \gamma^{++}_{11}\gamma^+_1\big)\big(1 + N_2\gamma^{+-}_{12}\gamma^-_2 + \gamma^{++}_{12}\gamma^+_2\big) + \\
N_1N_2\gamma^{+-}_{11}\gamma^{+-}_{12}\gamma^-_1\gamma^-_2\big(1 + (N_2-1)\gamma^{--}_{12}\gamma^{--}_{22}\gamma^-_2 + \gamma^{-+}_{12}\gamma^{+-}_{22}\gamma^+_2\big) + \\
N_2\gamma^{++}_{11}\gamma^{+-}_{12}\gamma^+_1\gamma^-_2\big(1 + (N_2-1)\gamma^{+-}_{12}\gamma^{--}_{22}\gamma^-_2 + \gamma^{++}_{12}\gamma^{+-}_{22}\gamma^+_1\big) + \\
N_2\gamma^-_2\big(1 + (N_1+1)\gamma^{+-}_{11}\gamma^{--}_{12}\gamma^-_1 + \gamma^{++}_{11}\gamma^{+-}_{12}\gamma^+_1\big) \cdot \\
\big(1 + (N_2-1)\gamma^{+-}_{12}\gamma^{--}_{22}\gamma^-_2 + \gamma^{++}_{12}\gamma^{+-}_{22}\gamma^+_2\big) + \\
\gamma^{++}_{12}\gamma^+_2\big(1 + (N_2+1)\gamma^{+-}_{22}\gamma^-_2 + \gamma^{++}_{22}\gamma^+_2\big) + \\
N_1\gamma^{+-}_{11}\gamma^{++}_{12}\gamma^-_1\gamma^+_2\big(1 + (N_2+1)\gamma^{--}_{12}\gamma^{+-}_{22}\gamma^-_2 + \gamma^{-+}_{12}\gamma^{++}_{22}\gamma^+_2\big) + \\
\gamma^{++}_{11}\gamma^{++}_{12}\gamma^+_1\gamma^+_2\big(1 + (N_2+1)\gamma^{+-}_{12}\gamma^{+-}_{22}\gamma^-_2 + \gamma^{++}_{12}\gamma^{++}_{22}\gamma^+_2\big) + \\
\gamma^+_2\big(1 + (N_1+1)\gamma^{+-}_{11}\gamma^{-+}_{12}\gamma^-_1 + \gamma^{++}_{11}\gamma^{++}_{12}\gamma^+_1\big) \cdot \\
\big(1 + (N_2+1)\gamma^{+-}_{12}\gamma^{+-}_{22}\gamma^-_2 + \gamma^{++}_{12}\gamma^{++}_{22}\gamma^+_2\big)\Big],
\end{aligned}
\tag{3}
$$

$$
\begin{aligned}
\langle S_1^- \rangle = \frac{\gamma_1^-}{Q}\Big[& N_1\big(1 + N_2\gamma_2^- + \gamma_2^+\big) + \\
& (N_1-1)N_1\gamma_{11}^{--}\gamma_1^-\big(1 + (N_2-1)\gamma_{12}^{--}\gamma_2^- + \gamma_{12}^{-+}\gamma_2^+\big) + \\
& N_1\big(1 + (N_1-1)\gamma_{11}^{--}\gamma_1^- + \gamma_{11}^{+-}\gamma_1^+\big)\big(1 + (N_2-1)\gamma_{12}^{--}\gamma_2^- + \gamma_{12}^{-+}\gamma_2^+\big) + \\
& (N_1+1)\gamma_{11}^{+-}\gamma_1^+\big(1 + N_2\gamma_{12}^{+-}\gamma_2^- + \gamma_{12}^{++}\gamma_2^+\big) + \\
& N_1N_2\gamma_{12}^{--}\gamma_{12}^{--}\gamma_2^-\big(1 + (N_2-1)\gamma_{22}^{--}\gamma_2^- + \gamma_{22}^{+-}\gamma_2^+\big) + \\
& (N_1-1)N_1N_2\gamma_{11}^{--}\gamma_{12}^{--}\gamma_1^-\gamma_2^-\big(1 + (N_2-1)\gamma_{12}^{--}\gamma_{22}^{--}\gamma_2^- + \gamma_{12}^{-+}\gamma_{22}^{+-}\gamma_2^+\big) + \\
& N_1N_2\gamma_2^-\big(1 + (N_1-1)\gamma_{11}^{--}\gamma_{12}^{--}\gamma_1^- + \gamma_{11}^{+-}\gamma_{12}^{+-}\gamma_1^+\big)\cdot \\
& \big(1 + (N_2-1)\gamma_{12}^{--}\gamma_{22}^{--}\gamma_2^- + \gamma_{12}^{-+}\gamma_{22}^{+-}\gamma_2^+\big) + \\
& (N_1+1)N_2\gamma_{11}^{+-}\gamma_{12}^{--}\gamma_1^+\gamma_2^-\big(1 + (N_2-1)\gamma_{12}^{+-}\gamma_{22}^{--}\gamma_2^- + \gamma_{12}^{++}\gamma_{22}^{+-}\gamma_2^+\big) + \\
& N_1\gamma_{12}^{-+}\gamma_2^+\big(1(N_2+1)\gamma_{22}^{+-}\gamma_2^- + \gamma_{22}^{++}\gamma_2^+\big) + \\
& (N_1-1)N_1\gamma_{11}^{--}\gamma_{12}^{-+}\gamma_1^-\gamma_2^+\big(1 + (N_2+1)\gamma_{12}^{--}\gamma_{22}^{+-}\gamma_2^- + \gamma_{12}^{-+}\gamma_{22}^{++}\gamma_2^+\big) + \\
& N_1\gamma_2^+\big(1 + (N_1-1)\gamma_{11}^{--}\gamma_{12}^{-+}\gamma_1^- + \gamma_{11}^{+-}\gamma_{12}^{++}\gamma_1^+\big)\cdot \\
& \big(1 + (N_2+1)\gamma_{12}^{--}\gamma_{22}^{+-}\gamma_2^- + \gamma_{12}^{-+}\gamma_{22}^{++}\gamma_2^+\big) + \\
& (N_1+1)\gamma_{11}^{+-}\gamma_{12}^{-+}\gamma_1^+\gamma_2^+\big(1 + (N_2+1)\gamma_{12}^{+-}\gamma_{22}^{+-}\gamma_2^- + \gamma_{12}^{++}\gamma_{22}^{++}\gamma_2^+\big)\Big],
\end{aligned}
\tag{4}
$$

$$
\begin{aligned}
\langle S_{11}^{++} \rangle = \frac{1}{Q}(\gamma_1^+)^2\gamma_{11}^{++}\Big[& 1 + N_2\gamma_{12}^{+-}\gamma_2^- + \gamma_{12}^{++}\gamma_2^+ + \\
& N_2\gamma_{12}^{+-}\gamma_2^-\big(1 + (N_2-1)\gamma_{12}^{+-}\gamma_{22}^{--}\gamma_2^- + \gamma_{12}^{++}\gamma_{22}^{+-}\gamma_2^+\big) + \\
& \gamma_{12}^{++}\gamma_2^+\big(1 + (N_2+1)\gamma_{12}^{+-}\gamma_{22}^{+-}\gamma_2^- + \gamma_{12}^{++}\gamma_{22}^{++}\gamma_2^+\big)\Big],
\end{aligned}
\tag{5}
$$

$$
\begin{aligned}
\langle S_{11}^{+-} \rangle = \frac{1}{Q}\gamma_1^+\gamma_1^-\gamma_{11}^{+-}\Big[& N_1\big(1 + (N_2-1)\gamma_{12}^{--}\gamma_2^- + \gamma_{12}^{-+}\gamma_2^+\big) + \\
& (N_1+1)\big(1 + N_2\gamma_{12}^{+-}\gamma_2^- + \gamma_{12}^{++}\gamma_2^+\big) + \\
& N_1N_2\gamma_{12}^{+-}\gamma_2^-\big(1 + (N_2-1)\gamma_{12}^{--}\gamma_{22}^{--}\gamma_2^- + \gamma_{12}^{-+}\gamma_{22}^{+-}\gamma_2^+\big) + \\
& (N_1+1)N_2\gamma_{12}^{--}\gamma_2^-\big(1 + (N_2-1)\gamma_{12}^{+-}\gamma_{22}^{--}\gamma_2^- + \gamma_{12}^{++}\gamma_{22}^{+-}\gamma_2^+\big) + \\
& N_1\gamma_{12}^{++}\gamma_2^+\big(1 + (N_2+1)\gamma_{12}^{--}\gamma_{22}^{+-}\gamma_2^- + \gamma_{12}^{-+}\gamma_{22}^{++}\gamma_2^+\big) + \\
& (N_1+1)\gamma_{12}^{-+}\gamma_2^+\big(1 + (N_2+1)\gamma_{12}^{+-}\gamma_{22}^{+-}\gamma_2^- + \gamma_{12}^{++}\gamma_{22}^{++}\gamma_2^+\big)\Big],
\end{aligned}
\tag{6}
$$

$$
\begin{aligned}
\langle S_{11}^{--} \rangle = \frac{N_1(N_1-1)}{Q}(\gamma_1^-)^2\gamma_{11}^{--}\Big[& 1 + (1-N_2)N_2(\gamma_{12}^{--})^2\gamma_{22}^{--}(\gamma_2^-)^2 + \\
& 2\gamma_{12}^{-+}\gamma_2^+ + (\gamma_{12}^{-+})^2\gamma_{22}^{++}(\gamma_2^+)^2 + \\
& \gamma_{12}^{--}\gamma_2^-\big(\gamma_{12}^{-+}\gamma_{22}^{+-}\gamma_2^+ - 1 + 2N_2(1 + \gamma_{12}^{-+}\gamma_{22}^{+-}\gamma_2^+)\big)\Big],
\end{aligned}
\tag{7}
$$

$$
\begin{aligned}
\langle S^{++}_{12} \rangle = \frac{1}{Q}\gamma^{+}_{1}\gamma^{+}_{2}\gamma^{++}_{12}\Big[& 2 + (2N_2+1)\gamma^{+-}_{22}\gamma^{-}_{2} + 2\gamma^{++}_{22}\gamma^{+}_{2} + \\
& \gamma^{++}_{11}\gamma^{+}_{1}\big(2 + (2N_2+1)\gamma^{+-}_{12}\gamma^{+-}_{22}\gamma^{-}_{2} + 2\gamma^{++}_{12}\gamma^{++}_{22}\gamma^{+}_{2}\big) + \\
& \gamma^{+-}_{11}\gamma^{-}_{1}\Big(1 + N_2\gamma^{--}_{12}\gamma^{+-}_{22}\gamma^{-}_{12} + \gamma^{-+}_{12}\gamma^{++}_{22}\gamma^{+}_{2} + \\
& N_1\big(2 + (2N_2+1)\gamma^{--}_{12}\gamma^{+-}_{22}\gamma^{-}_{2} + 2\gamma^{-+}_{12}\gamma^{++}_{22}\gamma^{+}_{2}\big)\Big)\Big],
\end{aligned}
\tag{8}
$$

$$
\begin{aligned}
\langle S^{+-}_{12} \rangle = \frac{\gamma^{+}_{1}\gamma_{2} - \gamma^{+-}_{12}}{Q}\Big[& N_2^2\gamma^{--}_{22}\gamma^{-}_{2}\big(2 + (2N_1+1)\gamma^{+-}_{11}\gamma^{--}_{12}\gamma^{-}_{1} + 2\gamma^{++}_{11}\gamma^{+-}_{12}\gamma^{+}_{1}\big) + \\
& \gamma^{+-}_{22}\gamma^{+}_{2}\big(1 + (N_1+1)\gamma^{+-}_{11}\gamma^{-+}_{12}\gamma^{-}_{1} + \gamma^{++}_{11}\gamma^{++}_{12}\gamma^{+}_{1}\big) + \\
& N_2\Big(- (2N_1+1)\gamma^{+-}_{11}\gamma^{-}_{1}\big(\gamma^{--}_{12}\gamma^{--}_{22}\gamma^{-}_{2} - 1 - \gamma^{-+}_{12}\gamma^{+-}_{22}\gamma^{+}_{2}\big) + \\
& 2\big(1 - \gamma^{--}_{22}\gamma^{-}_{2} + \gamma^{+-}_{22}\gamma^{+}_{2} + \gamma^{++}_{11}\gamma^{+}_{1}(1 - \gamma^{+-}_{12}\gamma^{--}_{22}\gamma^{-}_{2} + \gamma^{++}_{12}\gamma^{+-}_{22}\gamma^{+}_{2})\big)\Big)\Big],
\end{aligned}
\tag{9}
$$

$$
\begin{aligned}
\langle S^{--}_{12} \rangle = \frac{\gamma^{-}_{1}\gamma_{2} - \gamma^{--}_{12}}{Q}\Big[& N_1(N_2-1)\big(1 + (N_1-1)\gamma^{--}_{11}\gamma^{--}_{12}\gamma^{-}_{1} + \gamma^{+-}_{11}\gamma^{+}_{1}\big) + \\
& N_1(N_2-1)N_2\gamma^{--}_{22}\gamma^{-}_{2}\big(1 + (N_1-1)\gamma^{--}_{11}\gamma^{--}_{12}\gamma^{-}_{1} + \gamma^{+-}_{11}\gamma^{+-}_{12}\gamma^{+}_{1}\big) + \\
& N_1(N_2+1)\gamma^{+-}_{22}\gamma^{+}_{2}\big(1 + (N_1-1)\gamma^{--}_{11}\gamma^{-+}_{12}\gamma^{-}_{1} + \gamma^{+-}_{11}\gamma^{++}_{12}\gamma^{+}_{1}\big) + \\
& N_1N_2\big(1 + (N_2-1)\gamma^{--}_{22}\gamma^{-}_{2} + \gamma^{+-}_{22}\gamma^{+}_{2}\big) + \\
& (N_1-1)N_1N_2\gamma^{--}_{11}\gamma^{-}_{1}\big(1 + (N_2-1)\gamma^{--}_{12}\gamma^{--}_{22}\gamma^{-}_{2} + \gamma^{-+}_{12}\gamma^{+-}_{22}\gamma^{+}_{2}\big) + \\
& (N_1+1)N_2\gamma^{+-}_{11}\gamma^{+}_{1}\big(1 + (N_2-1)\gamma^{+-}_{12}\gamma^{--}_{22}\gamma^{-}_{2} + \gamma^{++}_{12}\gamma^{+-}_{22}\gamma^{+}_{2}\big)\Big].
\end{aligned}
\tag{10}
$$

There are a total of 14 observables, with those not described here found by swapping a and b in the indices of supplementary Eqs. 3-7 and 9. We solve the full system of 15 equations (including the partition function in supplementary Eq. 1) with the Mathematica function *NSolve*.

2.2 Comparison with Master and Langevin equations

Here, we compare our MaxCal approach with more traditional methods of model construction. The first of these is the master equation, in which the probability of the system occupying any given state or configuration is described for all time.

In order to write a master equation corresponding to the same shape-change process as described by our MaxCal model, we must include terms accounting for the same set of possible observable changes, that is all combinations of two or fewer changes in each principal component. A general model accounting for all possible events can then be trained on the rates with which each event is observed in the data. We have already recorded these rates as combinations of MaxCal γ values, and so can write a master equation as follows:

$$
\begin{aligned}
\frac{dP[N_1, N_2]}{dt} \quad = \quad & \gamma_1^+ \gamma_1^+ \gamma_{1\,1}^{++} \Big(2\gamma_2^+ \gamma_2^+ \gamma_{2\,2}^{++} \gamma_{1\,2}^{++} P[N_1 - 2, N_2 - 2] + \\
& 2\gamma_2^+ \gamma_{1\,2}^{++} P[N_1 - 2, N_2 - 1] + \\
& \big((2N_2 + 1)\gamma_2^+ \gamma_2^- \gamma_{2\,2}^{+-} \gamma_{1\,2}^{+-} \gamma_{1\,2}^{++} + 1\big) P[N_1 - 2, N_2] + \\
& 2(N_2 + 1)\gamma_2^- \gamma_{1\,2}^{+-} P[N_1 - 2, N_2 + 1] + \\
& 2(N_2 + 2)(N_2 + 1)\gamma_2^- \gamma_2^- \gamma_{2\,2}^{--} \gamma_{1\,2}^{+-} P[N_1 - 2, N_2 + 2] \Big) + \\
& \gamma_1^+ \Big(2\gamma_2^+ \gamma_2^+ \gamma_{2\,2}^{++} \gamma_{1\,2}^{++} P[N_1 - 1, N_2 - 2] + \\
& \gamma_2^+ (\gamma_{1\,2}^{++} + 1) P[N_1 - 1, N_2 - 1] + \\
& (2N_2 + 1)\gamma_2^+ \gamma_2^- \gamma_{2\,2}^{+-} \big(\gamma_{1\,2}^{+-} + \gamma_{1\,2}^{++}\big) P[N_1 - 1, N_2] + \\
& (N_2 + 1)\gamma_2^- (\gamma_{1\,2}^{+-} + 1) P[N_1 - 1, N_2 + 1] + \\
& 2(N_2 + 2)(N_2 + 1)\gamma_2^- \gamma_2^- \gamma_{2\,2}^{--} \gamma_{1\,2}^{+-} P[N_1 - 1, N_2 + 2] \Big) +
\end{aligned}
$$

$$\Bigg(\gamma_2^+ \gamma_2^+ \gamma_{2\,2}^{++} P[N_1, N_2 - 2] + 2\gamma_2^+ P[N_1, N_2 - 1] +$$
$$(2N_2 + 1)\gamma_2^+ \gamma_2^- \gamma_{2\,2}^{+-} P[N_1, N_2] +$$
$$2(N_2 + 1)\gamma_2^- P[N_1, N_2 + 1] +$$
$$(N_2 + 2)(N_2 + 1)\gamma_2^- \gamma_2^- \gamma_{2\,2}^{--} P[N_1, N_2 + 2] \Bigg) +$$
$$(2N_1 + 1)\gamma_1^+ \gamma_1^- \gamma_{1\,1}^{+-} \Bigg(2\gamma_2^+ \gamma_2^+ \gamma_{2\,2}^{++} \gamma_{1\,2}^{++} \gamma_{1\,2}^{-+} P[N_1, N_2 - 2] +$$
$$\gamma_2^+ (\gamma_{1\,2}^{++} + \gamma_{1\,2}^{+-}) P[N_1, N_2 - 1] +$$
$$((2N_2 + 1)\gamma_2^+ \gamma_2^- \gamma_{2\,2}^{+-} (\gamma_{1\,2}^{--} \gamma_{1\,2}^{++} + \gamma_{1\,2}^{+-} \gamma_{1\,2}^{-+}) + 1) P[N_1, N_2] +$$
$$(N_2 + 1)\gamma_2^- (\gamma_{1\,2}^{+-} + \gamma_{1\,2}^{--}) P[N_1, N_2 + 1] +$$
$$2(N_2 + 2)(N_2 + 1)\gamma_2^- \gamma_2^- \gamma_{2\,2}^{--} \gamma_{1\,2}^{+-} \gamma_{1\,2}^{--} P[N_1, N_2 + 2] \Bigg) +$$
$$(N_1 + 1)\gamma_1^- \Bigg(2\gamma_2^+ \gamma_2^+ \gamma_{2\,2}^{++} \gamma_{1\,2}^{-+} P[N_1 + 1, N_2 - 2] +$$
$$\gamma_2^+ (\gamma_{1\,2}^{-+} + 1) P[N_1 + 1, N_2 - 1] +$$
$$(2N_2 + 1)\gamma_2^+ \gamma_2^- \gamma_{2\,2}^{+-} (\gamma_{1\,2}^{--} + \gamma_{1\,2}^{-+}) P[N_1 + 1, N_2] +$$
$$(N_2 + 1)\gamma_2^- (\gamma_{1\,2}^{--} + 1) P[N_1 + 1, N_2 + 1] +$$
$$2(N_2 + 2)(N_2 + 1)\gamma_2^- \gamma_2^- \gamma_{2\,2}^{--} \gamma_{1\,2}^{--} P[N_1 + 1, N_2 + 2] \Bigg) +$$
$$(N_1 + 2)(N_1 + 1)\gamma_1^- \gamma_1^- \gamma_{1\,1}^{--} \Bigg(2\gamma_2^+ \gamma_2^+ \gamma_{2\,2}^{++} \gamma_{1\,2}^{-+} P[N_1 + 2, N_2 - 2] +$$
$$2\gamma_2^+ \gamma_{1\,2}^{-+} P[N_1 + 2, N_2 - 1] +$$
$$((2N_2 + 1)\gamma_2^+ \gamma_2^- \gamma_{2\,2}^{+-} \gamma_{1\,2}^{--} \gamma_{1\,2}^{-+} + 1) P[N_1 + 2, N_2] +$$
$$2(N_2 + 1)\gamma_2^- \gamma_{1\,2}^{--} P[N_1 + 2, N_2 + 1] +$$
$$2(N_2 + 2)(N_2 + 1)\gamma_2^- \gamma_2^- \gamma_{2\,2}^{--} \gamma_{1\,2}^{--} P[N_1 + 2, N_2 + 2] \Bigg) -$$
$$\Bigg[\gamma_1^+ \gamma_1^+ \gamma_{1\,1}^{++} \Bigg(2\gamma_2^+ \gamma_2^+ \gamma_{2\,2}^{++} \gamma_{1\,2}^{++} + 2\gamma_2^+ \gamma_{1\,2}^{++} + 1 +$$
$$(2N_2 + 1)\gamma_2^+ \gamma_2^- \gamma_{2\,2}^{+-} \gamma_{1\,2}^{++} \gamma_{1\,2}^{+-} + 2N_2 \gamma_2^- \gamma_{1\,2}^{+-} +$$
$$2N_2(N_2 - 1)\gamma_2^- \gamma_2^- \gamma_{2\,2}^{--} \gamma_{1\,2}^{+-} \Bigg) +$$
$$\gamma_1^+ \Bigg(2\gamma_2^+ \gamma_2^+ \gamma_{2\,2}^{++} \gamma_{1\,2}^{++} + \gamma_2^+ (1 + \gamma_{1\,2}^{++}) + 1 +$$
$$(2N_2 + 1)\gamma_2^+ \gamma_2^- \gamma_{2\,2}^{+-} (\gamma_{1\,2}^{++} + \gamma_{1\,2}^{+-}) + N_2 \gamma_2^- (1 + \gamma_{1\,2}^{+-}) +$$
$$2N_2(N_2 - 1)\gamma_2^- \gamma_2^- \gamma_{2\,2}^{--} \gamma_{1\,2}^{+-} \Bigg) +$$
$$\Bigg(\gamma_2^+ \gamma_2^+ \gamma_{2\,2}^{++} + 2\gamma_2^+ + 2N_2 \gamma_2^- + N_2(N_2 - 1)\gamma_2^- \gamma_2^- \gamma_{2\,2}^{--} \Bigg) +$$

$$
\begin{aligned}
&(2N_1+1)\gamma_1^{+}\gamma_1^{-}\gamma_{1\,1}^{+-}\Big(2\gamma_2^{+}\gamma_2^{+}\gamma_{2\,2}^{++}\gamma_{1\,2}^{++}\gamma_{1\,2}^{-+}+\gamma_2^{+}(\gamma_{1\,2}^{++}+\gamma_{1\,2}^{-+})+\\
&N_2\gamma_2^{-}(\gamma_{1\,2}^{+-}+\gamma_{1\,2}^{--})+2N_2(N_2-1)\gamma_2^{-}\gamma_2^{-}\gamma_{2\,2}^{--}\gamma_{1\,2}^{--}\gamma_{1\,2}^{+-}\Big)+\\
&N_1\gamma_1^{-}\Big(2\gamma_2^{+}\gamma_2^{+}\gamma_{2\,2}^{++}\gamma_{1\,2}^{-+}+\gamma_2^{+}(1+\gamma_{1\,2}^{-+})+1+\\
&(2N_2+1)\gamma_2^{+}\gamma_2^{-}\gamma_{2\,2}^{+-}(\gamma_{1\,2}^{-+}+\gamma_{1\,2}^{--})+N_2\gamma_2^{-}(1+\gamma_{1\,2}^{--})+\\
&2N_2(N_2-1)\gamma_2^{-}\gamma_2^{-}\gamma_{2\,2}^{--}\gamma_{1\,2}^{--}\Big)+\\
&N_1(N_1-1)\gamma_1^{-}\gamma_1^{-}\gamma_{1\,1}^{--}\Big(2\gamma_2^{+}\gamma_2^{+}\gamma_{2\,2}^{++}\gamma_{1\,2}^{-+}+2\gamma_2^{+}\gamma_{1\,2}^{-+}+1+\\
&(2N_2+1)\gamma_2^{+}\gamma_2^{-}\gamma_{2\,2}^{+-}\gamma_{1\,2}^{-+}\gamma_{1\,2}^{--}+N_2\gamma_2^{-}\gamma_{1\,2}^{--}+\\
&2N_2(N_2-1)\gamma_2^{-}\gamma_2^{-}\gamma_{2\,2}^{--}\gamma_{1\,2}^{--}\Big)\Big]P[N_1,N_2]. \qquad (11)
\end{aligned}
$$

This equation is arranged as follows: It is first divided into two sections. The first of these preceeds the square brackets $\Big[...\Big]$, and describes changes from another state into the state $[N_1, N_2]$. The second is contained within these square brackets, and describes the evolution out of the state $[N_1, N_2]$. Each of these sections is then divided into six major terms, each multiplied by the contents of large round brackets $\Big(...\Big)$. These terms, in order, represent a state change to N_1 from $N_1 - 2$, from $N_1 - 1$, no change in N_1, both an increase and a decrease in N_1, a state change to N_1 from $N_1 + 1$, and from $N_1 + 2$. The changes to N_2 are contained within the round brackets, and are ordered as follows: a change to N_2 from $N_2 - 2$, from $N_2 - 1$, both an increase and a decrease in N_2, no change in N_2, a state change to N_2 from $N_2 + 1$, and from $N_2 + 2$. The section in the square brackets is divided internally in the same way, though it is simpler as it represents changes that are all from the same initial state.

In merging the two time-steps, this master equation assumes that the system is Markovian (that is, the next event only depends on the current state of the system, not on its trajectory). In order to test this assumption, we ran Gillespie simulations corresponding to this master equation. We compared the correlations of trajectories from these simulations with those from real data, from which they diverged greatly (see main text Fig. 5E).

In order to see if the Markovian assumption was responsible for this divergence, we then developed a non-Markovian master equation, in which event probabilities depended on the last event to take place. This model need only account for a single time-step per event, as correlations with subsequent events are achieved through event-dependent state switching. The model has nine states, corresponding to the nine possible events that might have preceeded it, which are an increase a decrease or no change across two components. These states take the following forms:

$$
\begin{aligned}
\frac{dP[N_1,N_2]}{dt}_{++} = {} & \gamma_1^{+}\gamma_{1\,1}^{++}\gamma_{1\,2}^{++}\big(\gamma_2^{+}\gamma_{2\,2}^{++}\gamma_{1\,2}^{++}P[N_1-1,N_2-1]+ \\
& P[N_1-1,N_2]+(N_2+1)\gamma_2^{-}\gamma_{1\,2}^{+-}P[N_1-1,N_2+1]\big)+ \\
& \big(\gamma_2^{+}\gamma_{2\,2}^{++}\gamma_{1\,2}^{++}P[N_1,N_2-1]+P[N_1,N_2]+(N_2+1)\gamma_2^{-}\gamma_{2\,2}^{+-}\gamma_{1\,2}^{+-}P[N_1,N_2]\big)+ \\
& (N_1+1)\gamma_1^{-}\gamma_{1\,1}^{+-}\gamma_{1\,2}^{-+}\big(\gamma_2^{+}\gamma_{2\,2}^{++}\gamma_{1\,2}^{++}P[N_1+1,N_2-1]+ \\
& P[N_1+1,N_2]+(N_2+1)\gamma_2^{-}\gamma_{2\,2}^{+-}\gamma_{1\,2}^{+-}P[N_1+1,N_2+1]\big)- \\
& (\gamma_1^{+}\gamma_{1\,1}^{++}\gamma_{1\,2}^{++}+1+N_1\gamma_1^{-}\gamma_{1\,1}^{+-}\gamma_{1\,2}^{-+})(\gamma_2^{+}\gamma_{1\,2}^{++}\gamma_{2\,2}^{++}+1+N_2\gamma_2^{-}\gamma_{2\,2}^{+-}\gamma_{1\,2}^{+-})P[N_1,N_2]
\end{aligned}
\tag{12}
$$

$$
\begin{aligned}
\frac{dP[N_1,N_2]}{dt}_{+0} = {} & \gamma_1^{+}\gamma_{1\,1}^{++}\big(\gamma_2^{+}\gamma_{1\,2}^{++}P[N_1-1,N_2-1]+ \\
& P[N_1-1,N_2]+(N_2+1)\gamma_2^{-}\gamma_{1\,2}^{+-}P[N_1-1,N_2+1]\big)+ \\
& \big(\gamma_2^{+}\gamma_{1\,2}^{++}P[N_1,N_2-1]+P[N_1,N_2]+(N_2+1)\gamma_2^{-}\gamma_{1\,2}^{+-}P[N_1,N_2]\big)+ \\
& (N_1+1)\gamma_1^{-}\gamma_{1\,1}^{+-}\big(\gamma_2^{+}\gamma_{1\,2}^{++}P[N_1+1,N_2-1]+ \\
& P[N_1+1,N_2]+(N_2+1)\gamma_2^{-}\gamma_{1\,2}^{+-}P[N_1+1,N_2+1]\big)- \\
& (\gamma_1^{+}\gamma_{1\,1}^{++}+1+N_1\gamma_1^{-}\gamma_{1\,1}^{+-})(\gamma_2^{+}\gamma_{1\,2}^{++}+1+N_2\gamma_2^{-}\gamma_{1\,2}^{+-})P[N_1,N_2]
\end{aligned}
\tag{13}
$$

$$
\begin{aligned}
\frac{dP[N_1,N_2]}{dt}_{+-} = {} & \gamma_1^{+}\gamma_{1\,1}^{++}\gamma_{1\,2}^{+-}\big(\gamma_2^{+}\gamma_{2\,2}^{+-}\gamma_{1\,2}^{++}P[N_1-1,N_2-1]+ \\
& P[N_1-1,N_2]+(N_2+1)\gamma_2^{-}\gamma_{1\,2}^{+-}P[N_1-1,N_2+1]\big)+ \\
& \big(\gamma_2^{+}\gamma_{2\,2}^{+-}\gamma_{1\,2}^{++}P[N_1,N_2-1]+P[N_1,N_2]+(N_2+1)\gamma_2^{-}\gamma_{2\,2}^{--}\gamma_{1\,2}^{+-}P[N_1,N_2]\big)+ \\
& (N_1+1)\gamma_1^{-}\gamma_{1\,1}^{+-}\gamma_{1\,2}^{--}\big(\gamma_2^{+}\gamma_{2\,2}^{+-}\gamma_{1\,2}^{++}P[N_1+1,N_2-1]+ \\
& P[N_1+1,N_2]+(N_2+1)\gamma_2^{-}\gamma_{2\,2}^{--}\gamma_{1\,2}^{+-}P[N_1+1,N_2+1]\big)- \\
& (\gamma_1^{+}\gamma_{1\,1}^{++}\gamma_{1\,2}^{+-}+1+N_1\gamma_1^{-}\gamma_{1\,1}^{+-}\gamma_{1\,2}^{--})(\gamma_2^{+}\gamma_{1\,2}^{++}\gamma_{2\,2}^{+-}+1+N_2\gamma_2^{-}\gamma_{2\,2}^{--}\gamma_{1\,2}^{+-})P[N_1,N_2]
\end{aligned}
\tag{14}
$$

$$
\begin{aligned}
\frac{dP[N_1,N_2]}{dt}_{-0} = {} & \gamma_1^{+}\gamma_{1\,1}^{+-}\big(\gamma_2^{+}\gamma_{1\,2}^{-+}P[N_1-1,N_2-1]+ \\
& P[N_1-1,N_2]+(N_2+1)\gamma_2^{-}\gamma_{1\,2}^{--}P[N_1-1,N_2+1]\big)+ \\
& \big(\gamma_2^{+}\gamma_{1\,2}^{-+}P[N_1,N_2-1]+P[N_1,N_2]+(N_2+1)\gamma_2^{-}\gamma_{1\,2}^{--}P[N_1,N_2]\big)+ \\
& (N_1+1)\gamma_1^{-}\gamma_{1\,1}^{+-}\big(\gamma_2^{+}\gamma_{1\,2}^{++}P[N_1+1,N_2-1]+ \\
& P[N_1+1,N_2]+(N_2+1)\gamma_2^{-}\gamma_{1\,2}^{+-}P[N_1+1,N_2+1]\big)- \\
& (\gamma_1^{+}\gamma_{1\,1}^{++}+1+N_1\gamma_1^{-}\gamma_{1\,1}^{+-})(\gamma_2^{+}\gamma_{1\,2}^{++}+1+N_2\gamma_2^{-}\gamma_{1\,2}^{+-})P[N_1,N_2]
\end{aligned}
\tag{15}
$$

$$
\begin{aligned}
\frac{dP[N_1,N_2]}{dt}_{00} = {} & \gamma_1^{+}\big(\gamma_2^{+}P[N_1-1,N_2-1]+ \\
& P[N_1-1,N_2]+(N_2+1)\gamma_2^{-}P[N_1-1,N_2+1]\big)+ \\
& \big(\gamma_2^{+}P[N_1,N_2-1]+P[N_1,N_2]+(N_2+1)\gamma_2^{-}P[N_1,N_2]\big)+ \\
& (N_1+1)\gamma_1^{-}\big(\gamma_2^{+}P[N_1+1,N_2-1]+ \\
& P[N_1+1,N_2]+(N_2+1)\gamma_2^{-}P[N_1+1,N_2+1]\big)- \\
& (\gamma_1^{+}+1+N_1\gamma_1^{-})(\gamma_2^{+}+1+N_2\gamma_2^{-})P[N_1,N_2]
\end{aligned}
\tag{16}
$$

$$
\begin{aligned}
\frac{dP[N_1,N_2]}{dt}_{--} = \; & \gamma_1^{+}\gamma_{1\,1}^{+-}\gamma_{1\,2}^{+-}\big(\gamma_2^{+}\gamma_{2\,2}^{+-}\gamma_{1\,2}^{-+}P[N_1-1,N_2-1]+ \\
& P[N_1-1,N_2]+(N_2+1)\gamma_2^{-}\gamma_{1\,2}^{--}P[N_1-1,N_2+1]\big)+ \\
& \big(\gamma_2^{+}\gamma_{2\,2}^{+-}\gamma_{1\,2}^{-+}P[N_1,N_2-1]+P[N_1,N_2]+(N_2+1)\gamma_2^{-}\gamma_{2\,2}^{--}\gamma_{1\,2}^{--}P[N_1,N_2]\big)+ \\
& (N_1+1)\gamma_1^{-}\gamma_{1\,1}^{--}\gamma_{1\,2}^{--}\big(\gamma_2^{+}\gamma_{2\,2}^{+-}\gamma_{1\,2}^{-+}P[N_1+1,N_2-1]+ \\
& P[N_1+1,N_2]+(N_2+1)\gamma_2^{-}\gamma_{2\,2}^{--}\gamma_{1\,2}^{--}P[N_1+1,N_2+1]\big)- \\
& (\gamma_1^{+}\gamma_{1\,1}^{+-}\gamma_{1\,2}^{+-}+1+N_1\gamma_1^{-}\gamma_{1\,1}^{--}\gamma_{1\,2}^{--}) \\
& (\gamma_2^{+}\gamma_{1\,2}^{-+}\gamma_{2\,2}^{+-}+1+N_2\gamma_2^{-}\gamma_{2\,2}^{--}\gamma_{1\,2}^{--})P[N_1,N_2],
\end{aligned}
\tag{17}
$$

where the annotations +, 0 and − indicate a previous increase, no change, or decrease in a component, and the first and second annotations are indicative of such a change in N_1 and N_2, respectively. The additional three states are found by permuting 1 and 2 appropriately. Simulations based on this model recovered the distribution of correlations observed in the data (main text Fig. 5E). This indicates that the process can only be addressed by methods that acknowledge a dependency on past events.

We then attempted to construct a model using a Langevin equation, in which shape changes are decided by deterministic fields with some form of additive noise. We first tried to construct this from our initial master equation. As the master equation describes the probability of any given change, the deterministic part of the Langevin equation can be found by summing over all values of N_1 and N_2, i.e.

$$
\begin{aligned}
\frac{d\langle N_1\rangle}{dt} &= \sum_{N_1=0}^{\infty} N_1 \frac{dP[N_{1,2}]}{dt} \\
\frac{d\langle N_2\rangle}{dt} &= \sum_{N_2=0}^{\infty} N_2 \frac{dP[N_{1,2}]}{dt}
\end{aligned}
\tag{18}
$$

For now we disregard the noise. This approach leads to a further set of equations describing the dynamics of the averages:

$$
\begin{aligned}
\frac{d\langle N_1\rangle}{dt} \;=\;& \gamma_1^{+}\gamma_1^{+}\gamma_{1\,1}^{++}\Bigg(2\gamma_2^{+}\gamma_2^{+}\gamma_{2\,2}^{++}\gamma_{1\,2}^{++}\sum_1 (N_1+2)\sum_2 P[N_1,N_2] + \\
& 2\gamma_2^{+}\gamma_{1\,2}^{++}\sum_1 (N_1+2)\sum_2 P[N_1,N_2] + \\
& \gamma_2^{+}\gamma_2^{-}\gamma_{2\,2}^{+-}\gamma_{1\,2}^{+-}\gamma_{1\,2}^{++}\sum_1 (N_1+2)\sum_2 (2N_2+1)P[N_1,N_2] + \\
& \sum_1 N_1 \sum_2 P[N_1,N_2] + 2\gamma_2^{-}\gamma_{1\,2}^{+-}\sum_1 (N_1+2)\sum_2 N_2 P[N_1,N_2] + \\
& 2\gamma_2^{-}\gamma_2^{-}\gamma_{2\,2}^{--}\gamma_{1\,2}^{+-}\sum_1 (N_1+2)\sum_2 N_2(N_2-1)P[N_1,N_2]\Bigg) + \\
& \gamma_1^{+}\Bigg(2\gamma_2^{+}\gamma_2^{+}\gamma_{2\,2}^{++}\gamma_{1\,2}^{++}\sum_1 (N_1+1)\sum_2 P[N_1,N_2] + \\
& 2\gamma_2^{+}(\gamma_{1\,2}^{++}+1)\sum_1 (N_1+1)\sum_2 P[N_1,N_2] + \\
& \gamma_2^{+}\gamma_2^{-}\gamma_{2\,2}^{+-}\gamma_{1\,2}^{+-}\gamma_{1\,2}^{++}\sum_1 (N_1+1)\sum_2 (2N_2+1)P[N_1,N_2] + \\
& \sum_1 (N_1+1)\sum_2 P[N_1,N_2] + 2\gamma_2^{-}(\gamma_{1\,2}^{+-}+1)\sum_1 (N_1+1)\sum_2 N_2 P[N_1,N_2] + \\
& 2\gamma_2^{-}\gamma_2^{-}\gamma_{2\,2}^{--}\gamma_{1\,2}^{+-}\sum_1 (N_1+1)\sum_2 N_2(N_2-1)P[N_1,N_2]\Bigg) + \\
& \Bigg(\gamma_2^{+}\gamma_2^{+}\gamma_{2\,2}^{++}\sum_1 N_1\sum_2 P[N_1,N_2] + 2\gamma_2^{+}\sum_1 N_1\sum_2 P[N_1,N_2] + \\
& 2\gamma_2^{+}\gamma_2^{-}\gamma_{2\,2}^{+-}\sum_1 N_1\sum_2 (2N_2+1)P[N_1,N_2] + \\
& 2\gamma_2^{-}N_2\sum_1 N_1\sum_2 P[N_1,N_2] + \\
& \gamma_2^{-}\gamma_2^{-}\gamma_{2\,2}^{--}\sum_1 N_1\sum_2 N_2(N_2-1)P[N_1,N_2]\Bigg) +
\end{aligned}
$$

$$\gamma_1^+\gamma_1^-\gamma_{1\,1}^{+-}\Bigg(2\gamma_2^+\gamma_2^+\gamma_{2\,2}^{++}\gamma_{1\,2}^{++}\gamma_{1\,2}^{-+}\sum_1 N_1(2N_1+1)\sum_2 P[N_1,N_2]+$$
$$2\gamma_2^+(\gamma_{1\,2}^{++}+\gamma_{1\,2}^{+-})\sum_1 N_1(2N_1+1)\sum_2 P[N_1,N_2]+$$
$$\gamma_2^+\gamma_2^-\gamma_{2\,2}^{+-}(\gamma_{1\,2}^{--}\gamma_{1\,2}^{++}+$$
$$\gamma_{1\,2}^{+-}\gamma_{1\,2}^{-+})\sum_1 N_1(2N_1+1)\sum_2(2N_2+1)P[N_1,N_2]+$$
$$2\gamma_2^-(\gamma_{1\,2}^{+-}+\gamma_{1\,2}^{--})\sum_1(2N_1+1)N_1\sum_2(N_2)P[N_1,N_2]+$$
$$2\gamma_2^-\gamma_2^-\gamma_{2\,2}^{--}\gamma_{1\,2}^{+-}\gamma_{1\,2}^{--}\sum_1 N_1(2N_1+1)\sum 2N_2(N_2-1)P[N_1,N_2]\Bigg)+$$
$$\gamma_1^-\Bigg(2\gamma_2^+\gamma_2^+\gamma_{2\,2}^{++}\gamma_{1\,2}^{-+}\sum_1 N_1(N_1-1)\sum_2 P[N_1,N_2]+$$
$$2\gamma_2^+(1+\gamma_{1\,2}^{-+})\sum_1 N_1(N_1-1)\sum_2 P[N_1,N_2]+\sum_1 N_1(N_1-1)\sum_2 P[N_1,N_2]+$$
$$\gamma_2^+\gamma_2^-\gamma_{2\,2}^{+-}\gamma_{1\,2}^{--}\gamma_{1\,2}^{-+}\sum_1 N_1(N_1-1)\sum_2(2N_2+1)P[N_1,N_2]+$$
$$2\gamma_2^-(\gamma_{1\,2}^{--}+1)\sum_1 N_1(N_1-1)\sum_2(N_2)P[N_1,N_2]+$$
$$2\gamma_2^-\gamma_2^-\gamma_{2\,2}^{--}\gamma_{1\,2}^{--}\sum_1 N_1(N_1-1)\sum_2 N_2(N_2-1)P[N_1,N_2]\Bigg)+$$
$$\gamma_1^-\gamma_1^-\gamma_{1\,1}^{--}\Bigg(2\gamma_2^+\gamma_2^+\gamma_{2\,2}^{++}\gamma_{1\,2}^{-+}\sum_1 N_1(N_1-1)(N_1-2)\sum_2 P[N_1,N_2]+$$
$$2\gamma_2^+\gamma_{1\,2}^{-+}\sum_1 N_1(N_1-1)(N_1-2)\sum_2 P[N_1,N_2]+$$
$$\gamma_2^+\gamma_2^-\gamma_{2\,2}^{+-}\gamma_{1\,2}^{--}\gamma_{1\,2}^{-+}\sum_1 N_1(N_1-1)(N_1-2)\sum_2(2N_2+1)P[N_1,N_2]+$$
$$2\gamma_2^-\gamma_{1\,2}^{--}\sum_1 N_1(N_1-1)(N_1-2)\sum_2 N_2P[N_1,N_2]+$$
$$2\gamma_2^-\gamma_2^-\gamma_{2\,2}^{--}\gamma_{1\,2}^{--}\sum_1 N_1(N_1-1)(N_1-2)\sum_2 N_2(N_2-1)P[N_1,N_2]\Bigg)-$$

$$
\begin{aligned}
&\Bigg[\gamma_1^+\gamma_1^+\gamma_{1\,1}^{++}\Bigg(\big(2\gamma_2^+\gamma_2^+\gamma_{2\,2}^{++}\gamma_{1\,2}^{++} + 2\gamma_2^+\gamma_{1\,2}^{++} + 1\big)\sum_1 N_1 \sum_2 P[N_1, N_2] + \\
&\gamma_2^+\gamma_2^-\gamma_{2\,2}^{+-}\gamma_{1\,2}^{++}\gamma_{1\,2}^{+-}\sum_1 N_1 \sum_2 (2N_2+1)P[N_1, N_2] + 2\gamma_2^-\gamma_{1\,2}^{+-}\sum_1 N_1 \sum_2 N_2 + \\
&2\gamma_2^-\gamma_2^-\gamma_{2\,2}^{--}\gamma_{1\,2}^{+-}\sum_1 N_1 \sum 2N_2(N_2-1)P[N_1, N_2]\Bigg) + \\
&\gamma_1^+\Bigg((2\gamma_2^+\gamma_2^+\gamma_{2\,2}^{++}\gamma_{1\,2}^{++} + 2\gamma_2^+(1+\gamma_{1\,2}^{++}) + 1)\sum_1 N_1 \sum_2 P[N_1, N_2] + \\
&\gamma_2^+\gamma_2^-\gamma_{2\,2}^{+-}(\gamma_{1\,2}^{++} + \gamma_{1\,2}^{+-})\sum_1 N_1 \sum_2 (2N_2+1)P[N_1, N_2] + \gamma_2^-(1+\gamma_{1\,2}^{+-})\sum_1 N_1 \sum 2N_2 P[N_1, N_2] + \\
&2\gamma_2^-\gamma_2^-\gamma_{2\,2}^{--}\gamma_{1\,2}^{+-}\sum_1 N_1 \sum_2 N_2(N_2-1)P[N_1, N_2]\Bigg) + \\
&\Bigg((\gamma_2^+\gamma_2^+\gamma_{2\,2}^{++} + 2\gamma_2^+\sum_1 N_1 \sum_2 P[N_1, N_2] + 2\gamma_2^-\sum_1 N_1 \sum_2 N_2 + \gamma_2^-\gamma_2^-\gamma_{2\,2}^{--}\sum_1 N_1 \sum_2 N_2(N_2-1)\Bigg) + \\
&\gamma_1^+\gamma_1^-\gamma_{1\,1}^{+-}\Bigg((2\gamma_2^+\gamma_2^+\gamma_{2\,2}^{++}\gamma_{1\,2}^{++}\gamma_{1\,2}^{-+} + \gamma_2^+(\gamma_{1\,2}^{++} + \gamma_{1\,2}^{-+}))\sum_1 N_1(2N_1+1)\sum_2 P[N_1, N_2] + \\
&\gamma_2^-(\gamma_{1\,2}^{+-} + \gamma_{1\,2}^{--})\sum_1 N_1(2N_1+1)\sum_2 N_2 P[N_1, N_2] + \\
&2\gamma_2^-\gamma_2^-\gamma_{2\,2}^{--}\gamma_{1\,2}^{--}\gamma_{1\,2}^{+-}\sum_1 N_1(2N_1+1)\sum_2 N_2(N_2-1)P[N_1, N_2]\Bigg) + \\
&\gamma_1^-\Bigg((2\gamma_2^+\gamma_2^+\gamma_{2\,2}^{++}\gamma_{1\,2}^{-+} + 2\gamma_2^+(1+\gamma_{1\,2}^{-+}) + 1)\sum_1 N_1^2 \sum_2 P[N_1, N_2] + \\
&\gamma_2^+\gamma_2^-\gamma_{2\,2}^{+-}(\gamma_{1\,2}^{-+} + \gamma_{1\,2}^{--})\sum_1 N_1^2 \sum_2 (2N_2+1)P[N_1, N_2] + \gamma_2^-(1+\gamma_{1\,2}^{--})\sum_1 N_1^2 \sum_2 N_2 P[N_1, N_2] + \\
&2\gamma_2^-\gamma_2^-\gamma_{2\,2}^{--}\gamma_{1\,2}^{--}\sum_1 N_1^2 \sum_2 N_2(N_2-1)P[N_1, N_2]\Bigg) + \\
&\gamma_1^-\gamma_1^-\gamma_{1\,1}^{--}\Bigg((2\gamma_2^+\gamma_2^+\gamma_{2\,2}^{++}\gamma_{1\,2}^{-+} + 2\gamma_2^+\gamma_{1\,2}^{-+} + 1)\sum_1 N_1^2(N_1-1)\sum_2 P[N_1, N_2] + \\
&\gamma_2^+\gamma_2^-\gamma_{2\,2}^{+-}\gamma_{1\,2}^{-+}\gamma_{1\,2}^{--}\sum_1 N_1^2(N_1-1)\sum_2 (2N_2+1)P[N_1, N_2] + \\
&2\gamma_2^-\gamma_{1\,2}^{--}\sum_1 N_1^2(N_1-1)\sum_2 N_2 P[N_1, N_2] + \\
&2\gamma_2^-\gamma_2^-\gamma_{2\,2}^{--}\gamma_{1\,2}{}^{--}\sum_1 N_1^2(N_1-1)\sum_2 N_2(N_2-1)P[N_1, N_2]\Bigg)\Bigg],
\end{aligned}
$$

with a similar equation for $\langle dN_2/dt \rangle$ found by permuting 1 and 2 throughout. This equation is structured in the same way as Eq 11. However, this equation does not lead to a closed-form

solution. While some terms can be approximately closed, e.g $\sum_2 (2N_2 - 1) P[N_1, N_2] \approx 2\langle N_2 \rangle$, others do not without making further assumptions about the distribution of their lower-order moments, e.g. $\sum_1 N_1^2 (N_1 - 1) \approx \langle N_1^3 \rangle \neq \langle N_1 \rangle^3$. As our general motivation was to construct a model without such assumptions, we do not explore this avenue further.
